# Ageing begins early: developmental origins of ageing trajectories

**DOI:** 10.1098/rstb.2025.0326

**Published:** 2026-11-12

**Authors:** Alibek Moldakozhayev, Alec W. Eames, Wayne Mitchell, Albina Tskhay, Cecília G. de Magalhães, Juan J. Alba-Linares, Kejun Ying, Bohan Zhang, Sirui Zhang, Sharif Iqbal, Ali D. Yucel, Alexander Tyshkovskiy, Jeremy M. Van Raamsdonk, Vadim N. Gladyshev

**Affiliations:** ^1^ Division of Genetics, Department of Medicine, Brigham and Women's Hospital, Harvard Medical School, Boston, MA, USA; ^2^ Broad Institute of MIT and Harvard, Cambridge, MA, USA; ^3^ Department of Neurology and Neurosurgery, McGill University, Montreal, Quebec, Canada; ^4^ Department of Family Medicine, McGill University, Montreal, Quebec, Canada; ^5^ Division of Experimental Medicine, Department of Medicine, McGill University, Montreal, Quebec, Canada; ^6^ Metabolic Disorders and Complications, and Brain Repair and Integrative Neuroscience Programs, Research Institute of the McGill University Health Centre, Montreal, Quebec, Canada; ^7^ Nanomaterials and Nanotechnology Research Center (CINN), Spanish National Research Council (CSIC) - University of Oviedo, Oviedo 33011, Spain; ^8^ Department of Neurology and Neurological Sciences, Stanford University School of Medicine, Stanford, CA, USA; ^9^ Knight Initiative for Brain Resilience, Wu Tsai Neurosciences Institute, Stanford University, Stanford, CA, USA

**Keywords:** ageing, development, longevity

## Abstract

Ageing has traditionally been viewed as a process of late-life organismal deterioration, with interventions aimed at slowing ageing being administered after the ageing process has already begun. However, recent advances in quantifying ageing indicate that the process of molecular ageing starts as early as the end of gastrulation. Further, growing evidence suggests that the foundations of how different systems of an organism age are established during prenatal and postnatal development. Herein, we review the impact of early-life environmental conditions on disease susceptibility, adaptation, energy metabolism, microbiome and neurodevelopmental processes to ascertain how changes early in life can affect ageing-related features late in life. We also link growth, reproductive capacity and ageing by providing examples when the balance between longevity and reproduction is shifted towards a longer life through the growth axis at a cost of fecundity across species. We encourage researchers to investigate interventions aimed at mitigating ageing during the developmental period of life, with the important note that such interventions must be assessed with extra caution, given that many life-extending strategies may attenuate growth and reproduction, which may be detrimental for the organism during development.

This article is part of the theme issue ‘Developmental circuits in ageing’.

## 1. Introduction

While the beginning of life is an open topic for discussion [1], the end of life is more clearly defined: it ends when the organism dies. That final stage of life is preceded by ageing: a nearly universal process consisting of countless deleterious molecular transformations (i.e. damage) leading to a progressive loss of organismal functionality in the vast majority of multicellular biological systems [2–5]. Interestingly, a small number of multicellular species, including hydra (*Hydra vulgaris*), the jellyfish *Turritopsis dohrnii*, Blanding's turtles (*Emydoidea blandingii*), naked mole rats (*Heterocephalus glaber*), ocean quahog clams (*Arctica islandica*) and Great Basin bristlecone pines (*Pinus longaeva*), exhibit negligible senescence, that is, the progressive loss of functionality is not observed for those organisms, probably reflecting exceptional capacity of damage clearance, repair and maintenance systems [6]. The progressive loss of functionality in a typically ageing biological system can be partially driven by the environment and its changes, followed by the necessity to adapt and respond through appropriate adjustment of metabolism. Despite attempts to adapt, the damage is still constantly generated and accumulated owing to the inherent imperfection of biological functions. The degree of this imperfection is, in part, predetermined at the moment of conception, when two haploid genomes merge to form a new organism that inherits both beneficial and deleterious genetic variants from its parents. Individuals carrying a higher burden of protein-truncating variants experience earlier onset of chronic diseases and increased mortality risk [7], while centenarians, who have fewer loss-of-function mutations, maintain health better and have longer lives [8]. Some mutations are particularly detrimental to health and may result in an accelerated ageing phenotype. For example, progeria syndromes like Hutchinson-Gilford progeria syndrome (HGPS) arise from developmental splicing defects, generating progerin, a permanently truncated lamin A protein. Progerin disrupts nuclear lamina integrity, impairs DNA repair and recapitulates systemic ageing phenotypes, including atherosclerosis and lipodystrophy [9,10]. HGPS manifests accelerated ageing hallmarks such as growth retardation, alopecia, dermal thinning and vascular calcification within the first decade of life [11].

While germline mutations represent some of the earliest determinants of an organismal ageing trajectory, most are responsible for only a fraction of the damage accumulated during ageing. The imperfectness feature, woven into each molecular function of our body, introduces a far broader and more complex array of detrimental changes, affecting all biological systems in countless ways. The highly multifaceted nature of ageing has led to ongoing disagreement among researchers over its foundational principles—what ageing truly is and, perhaps equally important, when it actually begins [12,13]. Understanding when ageing begins is important from a practical standpoint, as any intervention aimed at slowing this process must be timed appropriately to be most effective. This review discusses the evidence that ageing begins during early development and shows that the trajectory of ageing is significantly influenced by the organism's early-life interactions with its environment.

## 2. The beginning of ageing

Ageing has traditionally been viewed as a gradual and irreversible process, recognized mainly through its later-life manifestations such as functional deterioration and the onset of chronic disease. The delayed onset of ageing-related decline has long misled the fundamental question of when ageing actually begins. The idea that development and ageing are intimately connected is not particularly new—Bidder was among the first to propose that aging begins where growth ends, and Williams' theory of antagonistic pleiotropy later formalized the notion that traits beneficial during development may become detrimental in later life [14,15]. Yet despite this long conceptual history and ongoing theoretical efforts to formalize these connections [16], a comprehensive synthesis of the empirical and epidemiological evidence linking specific developmental processes to ageing trajectories has remained lacking, which is the gap the present manuscript aims to address.

The emerging evidence suggests that ageing does not start in adulthood [17], or even after birth, but instead traces back to embryogenesis. Several recent studies using epigenetic and transcriptomic ageing clocks have revealed a natural rejuvenation event followed by ageing during early embryonic development, suggesting that ageing trajectories are already being established at the earliest stages of life [18–20]. In particular, this natural rejuvenation event was characterized by the predicted biological age decline shortly after fertilization, reaching its lowest point around gastrulation—defined as the Ground Zero of organismal ageing [21]. After this stage, biological age, defined as a cumulative burden of accumulated damage and estimated by molecular clocks and other measures of ageing progression, increases until an organism dies. The finding that ageing may begin as early as gastrulation raises the intriguing possibility that ageing-targeted interventions could, in principle, be applied during early development. In *Caenorhabditis elegans*, suppression of mitochondrial electron transport chain and ATP synthase activities exclusively during development results in an extension of adult lifespan [22]. Interestingly, restoring mitochondrial function in adulthood fails to reverse improved longevity, while the same intervention into mitochondrial function initiated during adulthood does not affect lifespan. However, this perturbation carries measurable side effects, including reduced body size and decreased behavioural rates, underscoring that developmental perturbations may have negative consequences. Consistent results have been obtained using pharmacological approaches: developmental exposure to cyrene was sufficient to extend lifespan and improve health in adult worms [23]. Whether analogous interventions could be translated to mammals remains highly speculative and requires extensive validation, given the considerably greater complexity and consequent vulnerability of mammalian development. At the same time, intervening only during the earliest stages of mammalian life can also influence long-term ageing trajectories and outcomes [24]. For example, when telomeres were artificially lengthened during early development, leading to the establishment of long telomeres across all cell types, the animals exhibited improved health, slower accumulation of cellular damage and extended lifespans relative to their wild-type counterparts.

## 3. The onset of diseases, development and ageing

In recent decades, mounting evidence has revealed the influence of early-life environmental conditions on ageing outcomes in adulthood, largely supporting the idea that adult diseases originate during development [25]. Preterm birth, defined as birth before 37 weeks of gestation, provides the most compelling evidence of this relationship—premature transition from *in utero* development results in underdeveloped organ systems, leading to increased risk of various age-related diseases, including hypertension [26], ischemic heart disease [27] and cerebrovascular disease [28] in adulthood. The relationship between gestational age at birth and ageing disease risk is inversely proportional: the earlier the preterm birth, the earlier the onset of chronic diseases in adulthood, with the poorest health outcomes observed in individuals born extremely preterm [29]. Since ageing is considered to be the primary cause for the development of age-related diseases [30], the dose-dependent relationship between the degree of developmental disruption and the onset of age-related diseases suggests that early alterations in development may accelerate the ageing process itself. At the same time, one alternative explanation may be that developmental disruptions may elevate the baseline mortality and disease risks without altering the ageing rate. Nevertheless, both explanations indicate that the trajectories of health and, potentially, ageing, are malleable during development.

Historical famines offer another sad opportunity to study the influence of early developmental perturbations on long-term health. The Dutch Famine of 1944–1945 stands as a particularly valuable natural experiment as it universally affected a well-defined population during a discrete period of food scarcity [31]. Individuals exposed to the famine, and specifically to restricted nutrition during *in utero* development, showed increased susceptibility to various age-related conditions in late life, including impaired glucose metabolism [32], coronary heart disease [33], diabetes [34] and obstructive airway disease [35]. The timing of developmental disruption was a critical factor: first-trimester exposure to famine resulted in worse health outcomes compared to exposure at a later time [31], suggesting that earlier developmental processes may influence the ageing trajectory more substantially. Additionally, organ-specific relationships were observed between development and disease. For instance, infants exposed to the famine during mid-gestation (the period of peak kidney nephron proliferation) were the only group to experience an increased risk of microalbuminuria, a marker of early kidney dysfunction, in adulthood [36], suggesting a direct link between perturbed organ development and the emergence of corresponding organ disease in adulthood. This exemplifies a broader principle that has recently received large-scale empirical support: organs can age at rates that diverge from whole-organism ageing trajectories, and accelerated organ-specific ageing is an increasingly recognized driver of organ-specific disease [37–39]. Developmental insults that compromise organ integrity or maintenance capacity from early life would therefore be expected to selectively accelerate ageing in the affected organ systems, manifesting as earlier onset of the corresponding diseases.

One possible cause for the impaired energy metabolism among individuals born during the Dutch Famine is maternal signalling about food scarcity in the external environment during early development. In response to this signalling, developmental programmes may have tuned organismal metabolism towards energy conservation strategies, including increased fat storage and higher insulin sensitivity. However, these individuals appeared to be maladapted to an environment of abundant nutrients, owing to their inability to effectively manage high glucose levels, which may have contributed to a subsequent earlier onset of age-related diseases. Therefore, early-life environmental exposures may play a critical role in shaping ageing trajectories by establishing long-term adaptive or maladaptive mechanisms (figure 1).

**Figure 1.**
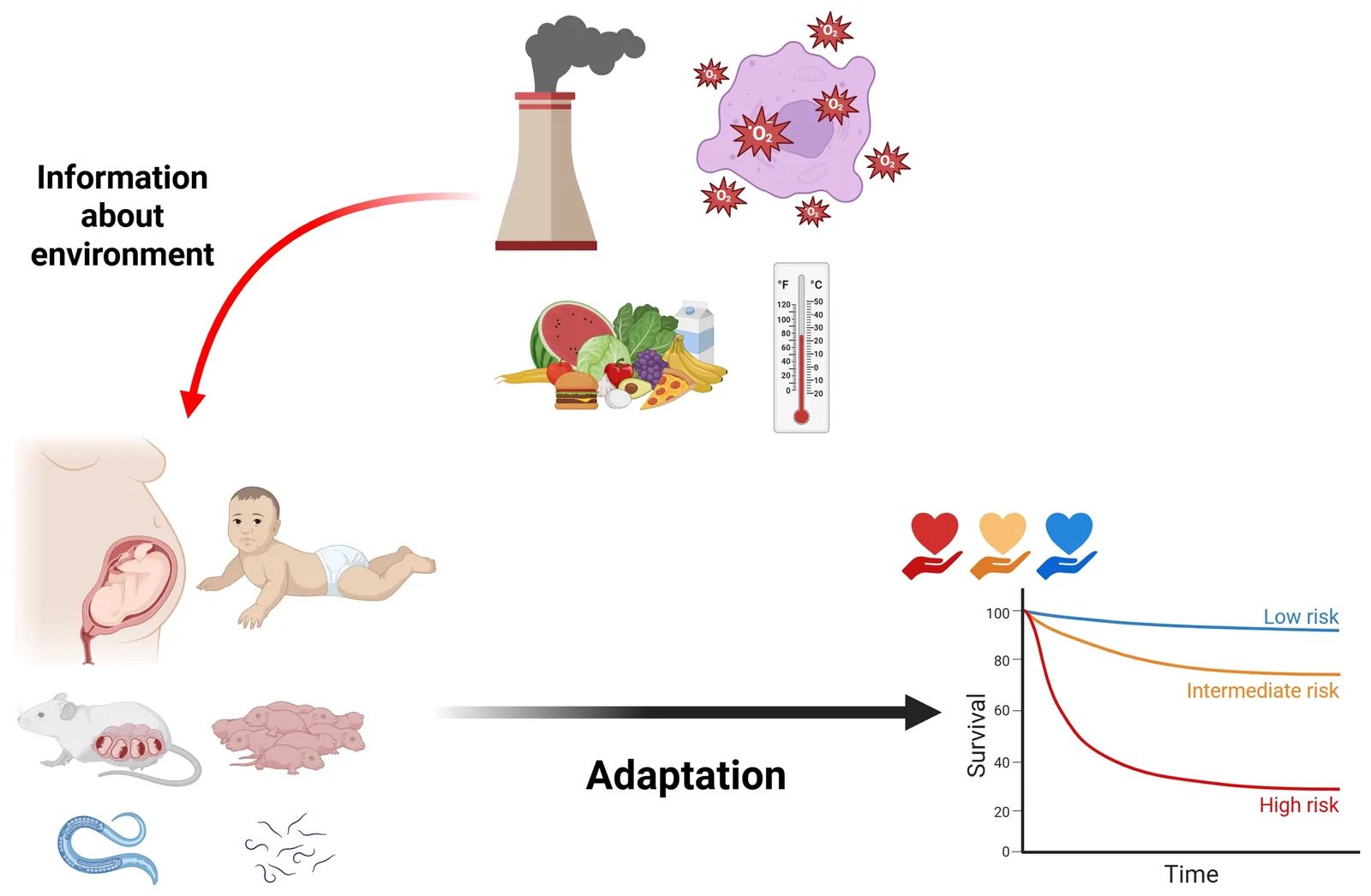
Early-life environmental exposures force the organism to adapt, and success in adaptation determines resilience, health and mortality risks during adulthood. Created in https://BioRender.com.

## 4. Adaptation, development and ageing

The idea that adaptation to the environment determines ageing outcomes is supported by the observation that transient, supposedly deleterious increases in reactive oxygen species (ROS) during early development in *C. elegans* were associated with enhanced stress resistance and extended lifespan in adulthood [40]. Interestingly, ROS variation during development established individualized stress resistance profiles by modulating global histone H3K4me3 levels, a mechanism confirmed in mammalian cells, thus suggesting the importance of epigenetic regulation in establishing early-life adaptive responses.

In mammals, postnatal development is a critical period in establishing epigenetic programmes that regulate cell differentiation [41–44], and recent research proposes that epigenetic alterations in early life may influence energy metabolism in adulthood [45,46]. In mice, maternal nutritional status was shown to induce lasting epigenetic modifications in the offspring that influenced metabolic regulation, health and ageing trajectories in later life. In particular, imbalances in maternal dietary patterns, such as a high-fat diet, have been shown to cause obesity and associated impairments in energy metabolism and health in their offspring [47]. Complementary to that, human studies demonstrated that the foundations of energy metabolism, specifically, increased brown adipose tissue activity leading to a leaner phenotype and lower body mass index, are also established early in development, as this phenotype was observed in individuals conceived during colder seasons [48].

The idea that energy metabolism is established during early life is further supported by the identification of DNA methylation signatures of childhood and adolescent obesity [49,50], body composition [49] and weight gain [51].

Throughout life, DNA methylation undergoes significant changes that are linked to lifespan regulation [52,53] and the onset of diseases [54–57]. This link has facilitated the development of predictive biomarkers [58], such as epigenetic clocks [59–66], to assess the pace of biological ageing, estimate risks of age-related diseases, morbidity and mortality, as well as the biological age itself. Interestingly, obesity was linked to higher epigenetic age acceleration in adults [67] and children [68]. Individuals having a higher pace of ageing even at birth had increased cardiometabolic risks, glycated haemoglobin levels, and more visceral fat during adolescence [69].

Notably, epigenetic clocks trained to predict chronological age capture the rate of changes for all kinds of molecular changes occurring over time throughout an organism's life. Such changes can be broadly categorized into three types: adaptive, neutral and deleterious. Recent work on causality-enriched epigenetic clocks has further clarified the distinction between adaptive and deleterious processes through the development of two separate epigenetic measures: DamAge, which captures the cumulative burden of molecular damage, and AdaptAge, which reflects adaptive changes happening to the organism [70]. The notion that adaptive processes follow a distinct biological trajectory from the accumulation of ageing-related damage has important implications for the design of longevity interventions. To be effective, such interventions must selectively target maladaptive or deleterious changes while preserving or even enhancing beneficial adaptations that contribute to resilience, which plays an important role in preserving longevity [71–73].

Together, these advances emphasize that signals captured by epigenetic clocks are partially shaped by environmental exposures that act early in life and persist across the lifespan. Among the most influential of these exposures is the microbiome, which interfaces directly with host epigenetic regulation, immune tone and metabolic homeostasis.

## 5. Microbiome, development and ageing

The human body is a mosaic of microbial and host interactions, with the gut microbiota emerging as a silent architect of health across the lifespan. Pioneering work by Elie Metchnikoff first hinted at the profound link between microbial communities and longevity, proposing that beneficial bacteria could delay ageing—a hypothesis now validated by modern science [74–77]. Early-life microbiota disruptions, whether from antibiotics, caesarean delivery, or environmental stressors, may cast a long shadow, shaping susceptibility to age-related diseases through inflammation, energy metabolism dysregulation and neuroimmune malfunction.

Antibiotic exposure in infancy, as demonstrated by Lynn *et al.* [78], provides the most striking example. Mice exposed to antibiotics developed divergent microbiota communities: a group of mice, dominated by *Lachnospiraceae* post-treatment, had shortened lifespans and neuroinflammation. Despite transient exposure to relatively safe antibiotics early in life, these mice exhibited chronic inflammation, impaired insulin signalling and microglial activation in the brain during adulthood. These outcomes mirror human studies linking caesarean sections, where infants experienced a disruption in microbial seeding have elevated body mass index and metabolic disease risks in later life [79]. Together, these findings underscore that early dysbiosis, even temporary, can irreversibly alter host physiology. Whether this reflects a permanently elevated baseline mortality and disease risk, an accelerated rate of ageing, or a combination of both, remains to be established. Regardless of which explanation predominates, both outcomes are undesirable, and both implicate early-life microbial seeding as a potentially modifiable determinant of long-term health trajectories, which may be intervened upon to improve ageing outcomes.

Similarly, Dinan & Cryan [74] highlight how gut-derived short-chain fatty acids regulate microglia and blood–brain barrier (BBB) integrity. Age-related BBB permeability increase, exacerbated by dysbiosis, permits neurotoxic metabolites to infiltrate the brain, potentially explaining the microglial hyperactivity and consequent neuroinflammation observed in Lynn's study. Consistent with this model, Parkinson's disease is characterized by a prodromal phase that can span decades and is frequently marked by early gut dysfunction, including dysbiosis and constipation, supporting the notion that microbial perturbations may initiate or amplify neurodegenerative processes via chronic disruption of the gut–brain axis [80].

The gut microbiota acts as a biological clock [81], and its early composition may influence the tempo for ageing. The mechanisms bridging early-life detrimental changes to microbiota and late-life disease may converge on systemic inflammation, barrier integrity and the development of a metabolic syndrome. The examples discussed in this chapter predominantly illustrate the consequences of harmful perturbations to the developing microbiome, and compelling examples of beneficial early-life microbiome-targeted interventions in the ageing context are currently lacking. We argue, however, that this reflects the nascent state of the field rather than an inherent impossibility. It is naturally easier to disrupt a complex biological system than to improve it, and large-scale assessments of perturbations confirm that while harmful effects predominate, a meaningful proportion of interventions do confer positive outcomes [82]. The microbiome represents an underexplored but mechanistically plausible target in this space, and identifying beneficial early-life microbial interventions is a goal that warrants dedicated investigation. Protecting microbial succession in infancy through judicious antibiotic use, dietary support, or targeted probiotics could defuse the inflammaging cascade, fostering resilience against metabolic, immune and neurodegenerative disorders. As Metchnikoff envisioned, nurturing our microbial allies may hold the key to a healthier, longer life.

## 6. Brain, development and ageing

Microbiota-driven processes directly intersect with brain development—a prolonged and highly plastic phase of human biology spanning from prenatal stages through early adulthood. Genetic and environmental factors that disrupt neural formation during this window may contribute to neurodevelopmental disorders [83–85] and alter ageing trajectories [86,87].

Brain development begins with a nearly uniform neuroepithelium formation, which undergoes extensive cell division to establish distinct layers and diverse neuronal populations [88–90]. During mid-gestation, rapid neurogenesis occurs, with neuronal precursor cells proliferating extensively between weeks 5 and 25 of human gestation [91,92]. The results of impaired neurogenesis, characterized by fewer proliferating cells and a lower number of neural progenitor cells compared to typically developing brains, can be observed in profound developmental disorders, such as Down syndrome (DS), caused by trisomy 21. DS individuals exhibit increased molecular age and mortality risk, reduced brain volume, along with disrupted cortical organization [20,93].

Beyond its impact on early brain development, impaired neurogenesis contributes to accelerated ageing, with early-onset Alzheimer's disease (AD) being one of the most significant consequences [94]. The foundations of neurodegenerative disorders, including AD, may be rooted in early-life dysregulation of the alternative splicing (AS) axis. AS is the process by which a single gene generates multiple protein isoforms and is a key factor underlying increased cellular and functional complexity in higher eukaryotes [95]. During embryonic development, the splicing factor PTBP1 suppresses the inclusion of neuron-specific exons (e.g. exon 10 of the MAPT gene encoding tau) to promote the proliferation of immature neurons [96]. In healthy maturation, PTBP1 is replaced by its paralogue PTBP2, enabling adult neurons to express exon 10-containing tau isoforms (4R-tau) with high microtubule-binding capacity. Failure to downregulate PTBP1 results in persistent embryonic splicing patterns (e.g. 3R-tau lacking exon 10), which exhibit reduced microtubule affinity and are prone to aggregation. These splicing dysregulations drive a pathological imbalance in tau isoform ratios (3R/4R-tau), which directly impair microtubule stability and nucleate neurofibrillary tangles. The resultant 3R-tau predominance phenocopies Alzheimer's-like tauopathy, correlating with progressive synaptic loss and cognitive deficits in both ageing models and human patients [97–99]. 3R-tau predominance is not exclusive to AD: it is also a defining neuropathological feature of Pick's disease, a distinct frontotemporal tauopathy [100]. Moreover, the splicing dysregulation mechanism described here is most directly relevant to early-onset AD, which accounts for approximately 5% of all AD cases and is more likely to involve strong genetic and developmental determinants than the late-onset form [101].

Lastly, X chromosome inactivation (XCI) in females is a striking example of how developmental mechanisms can shape long-term brain ageing. The brain is particularly dependent on genes located on the X chromosome, as many of these genes are highly expressed in neural tissues [102,103]. Alterations in X gene expression have been associated with neurodevelopmental and intellectual disabilities [104,105], highlighting the importance of X chromosome regulation for the brain. In females, random XCI during early development results in a mosaic pattern of cells expressing either the maternal (Xm) or paternal (Xp) X chromosome [106]. Recent findings suggest that skewing towards maternal X activation, as opposed to X mosaicism, can negatively influence the ageing process [107]. In a study where Xm was artificially enforced as the sole active X (Xm skew), female mice exhibited reduced cognitive performance in old age, as assessed by behavioural testing, and showed accelerated hippocampal ageing, measured through DNA methylation patterns. Intriguingly, in aged mice with mosaic X chromosome activation, neurons with an active Xm displayed a higher DNA methylation age compared to neighbouring neurons with an active Xp chromosome within the same individual. Complementing this picture, the stability of XCI itself appears to erode with age. A recent study using allele-specific single-nucleus RNA sequencing analysis demonstrated that ageing remodels transcription of the inactive X chromosome (Xi) across hippocampal cell types in female mice, with select Xi genes undergoing *de novo* escape from silencing, including *Plp1*, encoding a myelin component, whose expression was also elevated in the parahippocampuses of aged women [108]. Interestingly, adeno-associated virus-mediated elevation of Plp1 improved cognition in both aged male and female mice, suggesting that age-induced Xi escape may represent an adaptive mechanism contributing to the well-documented female advantage in cognitive resilience during ageing. Together, these results highlight how developmental processes such as XCI can have long-lasting effects on brain ageing, with the balance between XCI mosaicism, parental X skewing, and age-related Xi escape potentially modulating vulnerability to cognitive decline in a sex-specific manner.

Together, brain studies underscore a broader principle: developmental programmes during early life can impose long-term effects on ageing trajectories and age-related phenotypes. Beyond brain development, an analogous fundamental relationship between development and ageing emerges at the organismal level through the regulation of growth-controlling pathways.

## 7. The crosstalk of fecundity and ageing through the growth axis

Growth is a process tightly regulated by growth hormone (GH) signalling pathways. While essential during early life, sustained activation of GH signalling beyond the developmental period has been associated with pro-ageing effects and accelerated health decline [109,110]. This antagonistic role of GH signalling is further supported by long-lived Snell [111] and Ames [112] dwarf mouse strains, which have hormonal deficiencies in growth-related pathways, show reduced levels of circulating insulin, glucose and insuin-like growth factor-1 (IGF-1; [113]), and live 30%–50% longer than comparable strains. In addition, recent efforts employing Mendelian randomization have identified similar associations in humans, linking several single-nucleotide polymorphisms in the IGF-1, mechanistic target of rapamycin (mTOR), and growth hormone signalling pathways to age-related health outcomes [114].

Targeting the cornerstone of growth regulation, mTOR, with rapamycin, is one of the most promising and robust ways to extend life across model organisms [115]. Although rapamycin is typically administered during adulthood [116] or late life [117], its application restricted to the first 45 days of life extended median lifespan by approximately 10% in both genetically heterogeneous UM-HET3 male mice and *Daphnia magna*. This early-life intervention also led to reduced body size and prolonged healthspan, albeit causing a modest delay in female reproduction. Supporting these findings, another study showed that administering rapamycin during the postnatal period (either from days 4 to 30 or days 30 to 60) increased lifespan by 9.6% and 9.1%, respectively, in both male and female mice, as well as in *Drosophila melanogaster* [118]. Finally, a brief rapamycin pulse early in life extended the lifespan of female flies and mitigated age-related gut pathologies in both *Drosophila* and mice [119].

One of the most potent activators of growth signalling pathways is nutrient availability. In *C. elegans*, a lack of nutrients during early development results in a profound metabolic remodelling, growth arrest and activation of a worm's dauer state [120]. Dauer worms are stress-resistant and extremely long-lived, though their reproductive ability is absent until the food scarcity ends, signalling that the environment is again favourable for reproduction. In mice, the crowded litter model, which limits nutrient access during early postnatal development in mice, has demonstrated that early-life nutritional restriction can reduce growth rate, modulate energy expenditure, enhance insulin sensitivity and ultimately extend lifespan [121]. Early-life nutrition also appears to shape mice's reproductive parameters. For example, in mice, maternal calorie restriction has been associated with earlier pubertal onset and a shortened reproductive lifespan [122], as well as reduced ovarian follicle numbers and increased ovarian oxidative stress in adult offspring [123]. Similar patterns have been observed in a bovine model, where maternal undernutrition elevated maternal progesterone levels and led to a diminished ovarian reserve in offspring [124].

In humans, genetic and phenotypic traits associated with greater reproductive fitness have been strongly negatively correlated with lifespan as well [125]. Interestingly, girls born small for gestational age (SGA) have been shown to exhibit reduced uterine and ovarian volumes during adolescence, along with lower ovulation rates [126,127]. At the same time, children with SGA exhibit significantly lower DamAge and higher AdaptAge compared to those with normal birth weight, suggesting that this phenotype may result in improved longevity [70]. Together, these findings and the data from model organisms suggest that developmental constraints in reproduction via downregulation of growth hormone signalling pathways may elicit compensatory adaptive responses that may allow the organism to live longer (figure 2). These observations partially align with the pace-of-life hypothesis [128], which more broadly proposes that a slower developmental and reproductive tempo results in extended longevity—though we note that this relationship is probably not universal, as slower development or late reproduction driven by pathological causes would not necessarily confer longevity benefits.

**Figure 2.**
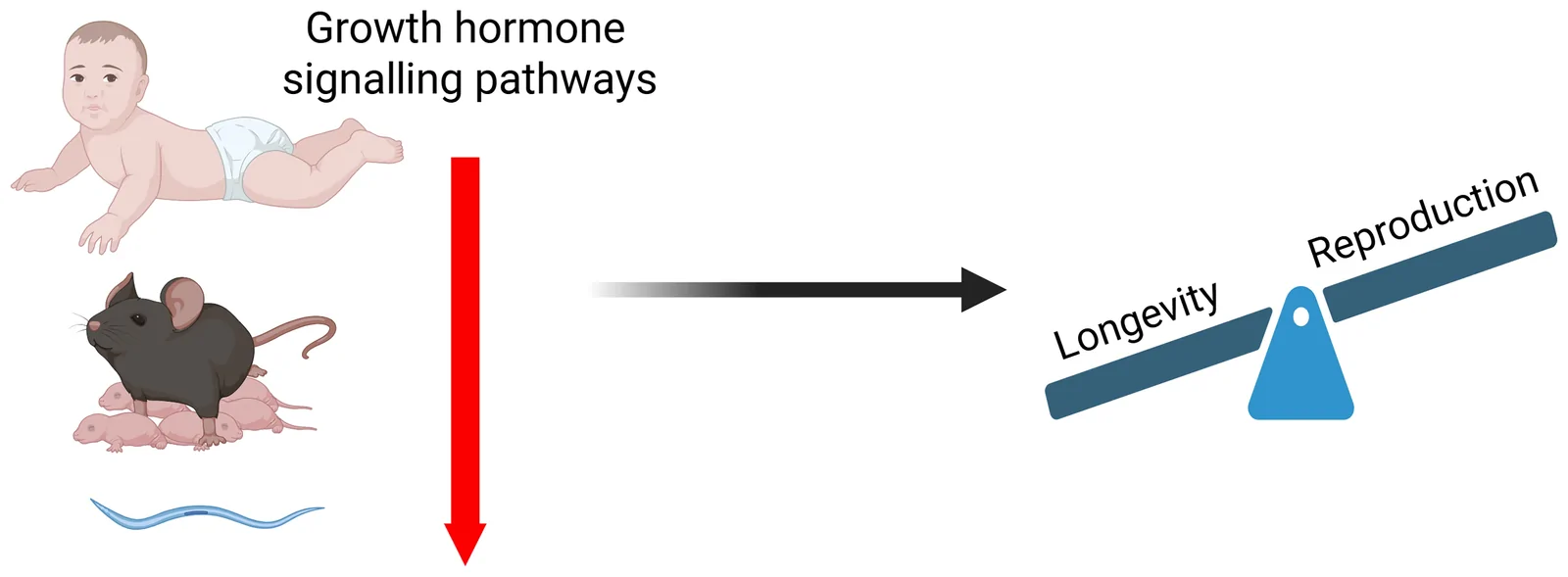
Downregulation of growth hormone signalling pathways during early development may result in improved longevity, potentially at the cost of reproduction. Created in https://BioRender.com.

## 8. Conclusions

There is accumulating evidence that ageing is not exclusively a late-life phenomenon but a lifelong process initiated early in development, following a ‘Ground Zero’ during gastrulation. From this point onwards, interactions between the developing organism and its environment shape molecular, cellular and physiological states that influence vulnerability to ageing-related decline later in life. Environmental stressors and adaptive demands during critical developmental windows can imprint long-lasting effects on ageing trajectories of individual organismal systems and an organism as a whole, yielding outcomes that are beneficial or detrimental depending on the nature, timing and intensity of these exposures.

Certain biological systems appear particularly sensitive to developmental perturbation. Protecting or optimizing these systems during early life by correcting neurodevelopmental errors, enhancing stress-response capacity, optimizing energy metabolism, preserving transcriptional fidelity, stabilizing the epigenome and fostering resilient host–microbiome interactions may reduce the lifelong accumulation of molecular damage and improve ageing outcomes. Importantly, such longevity-promoting adaptations may come at a cost. Shifts towards extended lifespan may incur trade-offs in other organismal functions, most notably reduced reproductive capacity, as observed in growth hormone–mediated lifespan extension across species.

These trade-offs warrant careful consideration, particularly in the context of early-life interventions. Most established geroprotective interventions, including caloric restriction, rapamycin and growth hormone receptor knockout, extend lifespan partly by suppressing biosynthetic and anabolic pathways—effects that are beneficial in the context of ageing but potentially disruptive during development, when these same pathways are essential for growth and organogenesis. Critically, the therapeutic window between beneficial and harmful effects is probably narrower during early life than in adulthood, given the heightened vulnerability of developing organisms to physiological changes. Any translational assessment of early-life geroprotective strategies in humans would therefore require rigorous preclinical validation across multiple model systems, with careful attention to dose, timing and developmental context. Importantly, extrapolating results from model organisms to unsupervised self-medication would be premature and potentially harmful.

Nevertheless, early-life interventions that prevent or correct developmental errors represent an underexplored strategy for mitigating future disease risk and modulating ageing trajectories. The clinical history of congenital heart disease (CHD) management provides a striking illustration of this principle. Whereas survival into adulthood was once rare, advances in early surgical intervention now allow over 90% of individuals with CHD to reach adulthood with ageing trajectories that closely resemble those of the general population [129]. This paradigm underscores the profound and lasting impact that developmental interventions can have on lifelong health and ageing, reinforcing the notion that the most effective strategies to promote longer and healthier lives may begin well before ageing manifestations become clinically apparent.

## Data Availability

This article has no additional data.

## Theme

One contribution of 21 to a theme issue ‘Developmental circuits in ageing’.
